# Use of Parabens (Methyl and Butyl) during the Gestation Period: Mitochondrial Bioenergetics of the Testes and Antioxidant Capacity Alterations in Testes and Other Vital Organs of the F1 Generation

**DOI:** 10.3390/antiox9121302

**Published:** 2020-12-18

**Authors:** Maria Manuel Oliveira, Fátima Martins, Mónica G. Silva, Elisete Correia, Romeu Videira, Francisco Peixoto

**Affiliations:** 1 Chemistry Research Centre (CQ-VR), University of Trás-os-Montes and Alto Douro (UTAD), 5000-801 Vila Real, Portugal; camelia05j@gmail.com (F.M.); m.g.27@live.com.pt (M.G.S.); 2 Center for Computational and Stochastic Mathematics (CEMAT), Department of Mathematics, IST-UL, Av. Rovisco Pais 1, 1049-001 Lisboa, Portugal; ecorreia@utad.pt; 3 REQUIMTE/LAQV, Laboratório de Farmacognosia, Departamento de Química, Faculdade de Farmácia, Universidade do Porto, R. Jorge Viterbo Ferreira, n. 228, 4050-313 Porto, Portugal; rvideira@ff.up.pt

**Keywords:** parabens, gestational exposure, mitochondrial bioenergetics, antioxidant activity, male infertility

## Abstract

Since the mid-1920s, parabens have been widely used as antimicrobial preservatives in processed foods and beverages, pharmaceuticals, and cosmetic products. Paraben use continues to generate considerable controversy, both in the general population and in the scientific community itself. The primary purpose of our study was to determine whether parabens (methyl and butyl at concentrations of 100 and 200 mg/kg body weight by subcutaneous injection) during pregnancy of adult female Wistar rats can have an impact on the F1 generation. As far as we know, we are the first to demonstrate that using parabens during pregnancy has negative repercussions on the mitochondrial bioenergetics and antioxidant activity of testicular germ cells in the F1 generation. Our study showed that there was a 48.7 and 59.8% decrease in the respiratory control index with 100 and 200 mg/kg of butylparaben, respectively. Cytochrome c oxidase activity was significantly inhibited (45 and 51%) in both groups. In addition, 200 mg/kg butylparaben promoted a marked decrease in citrate synthase activity, indicating that mitochondrial content decreased in the germ cells, especially spermatocytes and spermatids. Mitochondrial ROS production increased in groups exposed to parabens in a concentration-dependent manner, especially the butyl one (102 and 130%). The groups exposed to butylparaben showed an increase in superoxide dismutase (SOD) and catalase (CAT) activities, while glutathione reductase (GR) and glutathione *S*-transferase (GST) decreased. With methylparaben, only differences in SOD and GR were observed; for the latter, this only occurred with the highest concentration. The glutathione (GSH)/glutathione disulfide (GSSG) ratio did not undergo any significant change. However, there was a considerable increase in hydroperoxide content in animals exposed to butylparaben, with 100 and 200 mg/kg resulting in 98.6 and 188% increase, respectively. Furthermore, several other organs also showed alterations in antioxidant capacity due to paraben use. In summary, our study demonstrates that paraben use during pregnancy will cause severe changes in the mitochondrial bioenergetics and antioxidant capacity of testicular germ cells and the antioxidant capacity of several other F1 generation organs.

## 1. Introduction

A significant proportion of the population is exposed daily to parabens due to the use of these types of compounds by the food, cosmetic, and pharmaceutical industries. Owing to the possible impact on human health, namely its implication in the induction of oxidative stress and reproductive health of current and future generations, parabens are considered as emerging toxic contaminants.

Parabens are structurally similar to alkylphenols and mimic the natural estrogen 17-β-estradiol by binding to the estrogenic receptor and influencing the expression of estrogen-dependent genes [1]. The most widely used parabens are methylparaben (MP), ethylparaben (EP), propylparaben (PP), and butylparaben (BP). MP and BP have the lowest and highest toxic and estrogenic potential, respectively [2]. However, parabens exhibit a much weaker affinity to estrogenic receptors compared to 17-β-estradiol [3]. Due to the widespread use of parabens in various consumer products, it is already possible to observe bioaccumulation of these chemicals in aquatic environments. Methylparaben was found in 100% of water and sediment samples and in almost 90% of fish, invertebrates, and plants. 4-hydroxybenzoic acid (4-HB) was the most abundant metabolite, having been found in 97% of the biotic and abiotic samples analyzed [4]. One study showed that paraben exposure was positively associated with the trimester gestational weight gain rate during pregnancy [5].

Several in vitro and in vivo studies on animal models have been published in recent decades examining the effects of parabens on oxidative stress and infertility. Many of these studies are related to male rodent infertility and have shown quite adverse effects on sperm count, testosterone levels, and reproductive organ weight after dietary exposure [6,7].

Regarding the F1 generation, a significant reduction in the body weight of female offspring and a significant reduction in the mass of testes, seminal vesicles, and prostate of male offspring were observed after maternal exposure to BP, administered subcutaneously at doses of 100 and 200 mg/kg [8]. Perinatal exposure to BP induced several alterations in the reproductive system of the male F1 generation [9].

Oral administration of relatively low doses (40, 20, and 13.3 mg/0.2 mL oil/kg body weight/day) of BP for 30 days resulted in a marked increase in lipid peroxidation and a reduction of nonenzymatic antioxidants, such as glutathione (GSH) and ascorbic acid content. A decrease in enzymatic antioxidants, such as superoxide dismutase (SOD), catalase (CAT), glutathione peroxidase (GP), and glutathione *S*-transferase (GST), was also observed [10].

The induction of oxidative stress by parabens was unequivocal with the involvement of MP (the least toxic of parabens) in the production of glutathione hydroquinone and glutathione–benzoquinone conjugates by reaction with oxygen singlet (1O_2_) and GSH as well as in the production of hydrogen peroxide [11,12]. These data are consistent with those obtained by Handa et al. (2006) and Lakeram et al. (2007), which showed that MP in the presence of ultraviolet B (UVB) significantly increased cell death, oxidative stress, nitric oxide (NO) production, and lipid peroxidation in HaCaT keratinocytes [13,14]. An increase in ROS has also been observed in sperm exposed to a paraben mixture [15].

Before being excreted in the urine, parabens are mainly metabolized in the liver and, to a lesser extent, in other organs [14]. However, a considerable number of studies have demonstrated the presence of nonmetabolized parabens in human body fluids and tissues.

Parabens harm the mitochondria [16], affecting the cells in both the germinative and vegetative phases [17]. Given the importance of mitochondria in all cells and spermatozoa, disorders in these organelles will have detrimental consequences on male fertility [18]. Because women are the group most exposed to parabens due to the extensive use of cosmetics, even during the gestational period, we aimed to evaluate the effect of MP and BP administration during gestation on the antioxidant capacity of various organs and the mitochondrial bioenergetics of testes of the male F1 generation.

## 2. Materials and Methods

### 2.1. Chemical Reagents

All chemicals used in this study were purchased from Sigma-Aldrich unless stated otherwise and were equal to or above analytical grade.

### 2.2. Animals and Treatments

**Figure 1:**
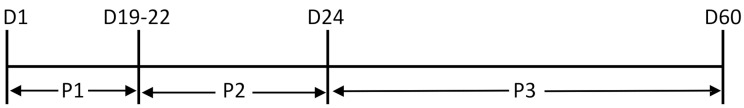
Experimental protocol. D1, first gestational day and beginning of paraben injection; D19–22 last gestational day and ending of paraben injection; D24, last lactational day and separation of males from females; D60, euthanasia; P1, gestational period; P2, lactational period; P3, postlactation period.

Male and female Wister rats were obtained from Charles River (Barcelona, Spain) and maintained under conditions of controlled lighting (lights on, 07.00–19.00), temperature of 19–21 °C, and humidity of 45–55%. The rats were fed a standard commercial pellet diet (20185 Teklad Global 18% protein rodent diet, sterilized), and tap water was provided ad libitum. Animal experiments began after an acclimation period of one week after arrival. To obtain pregnant animals, virgin females aged 14–17 weeks and weighing 200 g were placed with males weighing 220–240 g. Three females and one male were housed together in a cage. The stage of the estrous cycle was determined based on vaginal cytology. Mating was confirmed by the presence of sperm in vaginal smears (gestational day 0), and the females were then separated from the males. The pregnant Wistar female rats were individually housed in cages organized in a 2-D array with five columns (groups) and 7–10 lines (n = 7–10 females/group) to evaluate the possible effects of treatment in the male offspring. Five groups-control (CTRL), 100 mg/kg methylparaben (MP-100), 200 mg/kg methylparaben (MP-200), 100 mg/kg butylparaben (BP-100), and 200 mg/kg butylparaben (BP-200)-were randomized using the website http://www.random.org. The paraben solutions were prepared in pure peanut oil so that the injected volume was the same in all groups, with the CTRL group receiving the same vehicle volume. From the beginning and throughout the gestation period (19–22 days), females were submitted to daily administration of parabens (MP or BP at concentrations of 100 and 200 mg/kg body weight) by subcutaneous injection, as shown in Figure 1. Maternal body weight was measured every three days during the treatment period to determine volume administration and signs of toxicity. The dose of 100 and 200 mg/kg was chosen based on several studies already published and considering the exposure to n-BP during gestation and lactation has adverse effects on the reproductive system of male offspring with no observed adverse effect level (NOAEL) of 160 mg/kg/day [8,19,20,21].

The experimental protocols and procedures were approved by the University of Trás-os-Montes and Alto Douro University ORBEA authorities and followed the Portuguese (Decreto-Lei 113, August 7th) and European (EU Directive 2010/63/EU) legislation.

During this period, animals were carefully observed for malaise signs. The body mass of each female was recorded daily to calculate the volume of each compound to be administered. Food and water consumption were also monitored daily.

Twenty-one days after birth, the litter was weaned, weighed, and separated from the progenitors. Males and females were divided based on evaluation of the anogenital distance as well as visualization of the scrotum.

To study the growth and development of males, the F1 generation was monitored twice a week by feed/water consumption and body mass. When they reached sexual maturity, approximately two months after birth, all males were sacrificed by cervical dislocation followed by decapitation and exsanguination. The organs (liver, testicles, seminal vesicles, kidneys, and heart) were collected, weighed, packed in prelabeled packages, and stored at −70 °C for enzymatic evaluation and oxidative stress parameters. For mitochondrial bioenergetics evaluation, rats were sacrificed and mitochondrial isolation was immediately performed.

### 2.3. Testis Mitochondria Isolation

Mitochondria were isolated from rat testes by standard differential centrifugation according to standardized methods with minor modifications. Briefly, the organ was removed, washed, and homogenized in an ice-cold homogenization medium containing sucrose (250 mM), HEPES (10 mM) at pH 7.4, EDTA (0.5 mM), and bovine serum albumin (BSA, 0.1%) without fat. EGTA and bovine serum albumin were removed from the final wash medium adjusted to pH 7.4. The tissue and cores were separated by centrifugation at 800× *g* for 10 min at 4 °C (Sigma 2K-16). Mitochondria were recovered from the supernatant by centrifugation at 10,000× *g* for 10 min at 4 °C. The brown mitochondrial sediment was washed twice, resuspended in wash medium, and divided into two aliquots: one was immediately used and the other was frozen at −80 °C to determine the activity of the complexes alone. Total protein content was established by the biuret method [22] calibrated with delipidated bovine serum albumin.

### 2.4. Mitochondria Respiration

The oxygen consumption of isolated liver mitochondria was monitored polarographically with a Clark-type oxygen electrode at 25 °C in a 1 mL water thermostatic incubation chamber (CB1-D Hansatech) with continuous agitation. The traditional reaction medium consisted of sucrose (130 mM), KCl (50 mM), MgCl_2_ (5 mM), KH_2_PO_4_ (5 mM), and HEPES (5 mM) adjusted to pH 7.2 on a 1 mg mitochondrial protein basis by 1 mL of buffer. After 2 min equilibration period, mitochondria were energized with succinate (5 mM), a substrate, in the presence of rotenone (3 μM) to avoid the reverse flow of electrons from complex II to complex I. State 4 respiration corresponded to resting oxygen consumption after phosphorylation of a small amount of Nicotinamide adenine dinucleotide, reduced form (ADP). State 3 was initiated by adding ADP (100 nmol mg protein^−1^). Respiratory control index (RCI) was measured as the ratio between the oxygen consumption rate in state 3 and state 4 [23].

### 2.5. Mitochondrial Membrane Potential (ΔΨ)

Mitochondrial transmembrane potential (ΔΨ) was measured by a fluorimetric method using safranin O dye [24] and recorded in a Varian Cary Eclipse fluorescence spectrophotometer (Varian Inc., Palo Alto, CA, USA) operating at 485 nm excitation and 586 nm emission at 30 °C. Freshly isolated mitochondria (0.5 mg protein) were suspended in 3 mL reaction medium containing sucrose (130 mM), KCl (50 mM), MgCl_2_ (5 mM), KH_2_PO_4_ (5 mM), and HEPES (5 mM) at pH 7.2 in the presence of safranine O dye (5 μM) supplemented with rotenone (1 μg·mL^−1^). Accumulation of safranine O dye in mitochondria is driven by membrane potential, resulting in decreased fluorescence, and mitochondrial depolarization results in the release of safranine O dye from mitochondria. The data obtained were calibrated using a K^+^ gradient. The fluorescence of safranin O was recorded in the presence of 2 nM valinomycin and a gradual increase in K^+^ concentration (0.2–120 mM), which allowed calculation of the transmembrane potential difference (Δψ) through the equation of Nernst assuming a matrix with [K^+^] = 150 mM.

### 2.6. Determination of Mitochondrial Enzyme Activity

All assays were carried out using 10 μg mitochondrial protein on a temperature-controlled Biotek Power Wave XS2 plate reader (Biotek Instruments, Winooski, VT, USA (30 °C) according to previously described methods [23]. Aliquots of mitochondrial suspensions were subjected to three freeze–thaw cycles to break intact mitochondria, and measurements were initiated by the addition of mitochondrial protein. Complex I (NADH: ubiquinone oxidoreductase) activity was monitored following the oxidation of NADH at 340 nm (ε = 6220 M^−1^ cm^−1^) in a medium containing potassium phosphate (50 mM) at pH 7.4, KCN (2 mM), MgCl_2_ (5 mM), BSA (2.5 mg mL^−1^), antimycin (0.03 mM), decylubiquinone (0.1 mM), and NADH (0.3 mM) to start the reaction. Only rotenone-sensitive activity was considered. Complex IV (cytochrome c oxidase) activity was determined at 550 nm in a microplate reader (Bio-Tek Instruments, Winooski, VT, USA). Enzyme assays were performed at 30 °C in a standard reaction medium containing Tris-HCl buffer (10 mM) and KCl (120 mM) at pH 7.0. The reaction was started by the addition of 20 μL of reduced cytochrome c (0.016 mM), and the slope was recorded for 30 s before and after KCN (100 mM). Cytochrome c was previously prepared by the reduction of cytochrome c with excess sodium dithionite [25]. The activity of the IV complex was expressed as nmol of cytochrome c oxidized·min^−1^·mg of protein^−1^.

Citrate synthase activity was determined following the reduction of 5,5′-dithio-bis (2-nitrobenzoic acid) (ε = 13,600 M^−1^ cm^−1^) in a buffer containing Tris-HCl (200 mM) at pH 8.0, 5,5-dithio-bis-(2-nitrobenzoic acid) (DTNB) (0.01 mM), Triton X-100 (0.02%), oxaloacetate (1 mM) and Acetil-CoA (0.37 mM). Results were expressed as citrate synthase·min^−1^·mg protein^−1^ [26].

### 2.7. Mitochondrial ROS Production

Mitochondrial ROS production was evaluated in fresh isolated mitochondria (0.5 mg /mL) and incubated at 30 °C in reaction buffer containing KH_2_PO_4_ (5 mM), EGTA (0.1 mM), MgCl_2_ (3 mM), KCl (145 mM), and HEPES (30 mM) at pH 7.4. The medium was supplemented with rotenone (2 μM), succinate (10 mM), homovanilic acid (100 μM), and horseradish peroxidase (3 U/mL). After 15 min of incubation, the reaction was stopped by adding 500 μL of glycine buffer (2 M) at pH 12, containing EDTA (25 mM), and the reaction mixture was transferred to ice. The sample was then centrifuged at 9000× *g* for 5 min, and the supernatant was transferred to a 3 mL quartz cell. Fluorescence of the supernatant was read at an excitation wavelength of 312 nm and emission of 420 nm in a Varian Cary Eclipse. The concentration of hydrogen peroxide was determined based on a standard curve of hydrogen peroxide.

### 2.8. Determination of Antioxidant Enzymes

SOD activity was assayed by measuring its ability to inhibit the reduction of nitrobluetetrazolium (NBT) at 560 nm [27] in the presence of potassium phosphate buffer (100 mM) at pH 7.4, EDTA (10 mM), NBT (10 mM), hypoxanthine (10 mM), and xanthine oxidase (0.023 U mol^−1^). One SOD unit was defined as the enzyme activity that inhibited the reduction of NBT to water-insoluble blue-colored formazan by 50%. CAT activity was measured by following oxygen production in the system using Clark’s oxygen electrode (Hansatech^®^) [28]. The reaction medium comprised potassium phosphate buffer (50 mM) at pH 7.0 and H_2_O_2_ (1 M) in a final volume of 1 mL. The medium buffer was previously subjected to a nitrogen stream to decrease the dissolved oxygen. After 2 min of thermostatic incubation at 30 °C and stabilization, H_2_O_2_ was added to the reaction medium. CAT activity was calculated as μmol H_2_O_2_/min/mg of protein. GR activity was performed using a Varian Cary 50 spectrophotometer according to the method described in [29]. The reaction medium consisted of potassium phosphate buffer (100 mM KH_2_PO_4_ and 0.5 mM EDTA, pH 7.4), oxidized glutathione (100 mM), and NADPH (10 mM). The reaction was started 2 min after thermostabilization by the addition of glutathione disulfide (GSSG). Enzyme activity was measured at 340 nm at 30 °C by NADPH oxidation. The result was expressed as mM NADPH oxidized/min/mg of protein (ε = 6220 M^−1^ cm^−1^). GST activity was determined by a Varian Cary 50 spectrophotometer [30]. The reaction medium contained potassium phosphate buffer (100 mM) at pH 7.0, 1-Chloro-2,4-dinitrobenzene (CDNB) (100 mM), 10% Triton X-100 (10% *v*/*v*), and GSH (100 mM) in a final volume of 2 mL. The reaction was initiated with the addition of GSH (100 mM). The absorbance variation was performed at 340 nm, and enzyme activity was expressed as mM CDNB conjugated/min/mg of protein (ε = 9600 M^−1^ cm^−1^).

### 2.9. Statistical Analysis

Descriptive statistics of data are presented as mean (M) and standard deviation (SD) when appropriate. Skewness and kurtosis coefficients were computed for univariate normality analysis purposes, and all values were within ±2. To determine if the administration of MP and BP had a statistically significant effect on testis mitochondrial bioenergetics and antioxidant capacity in several organs, a multivariate analysis of variance (MANOVA) was performed, followed by one-way analysis of variance (ANOVA) and post hoc tests, when appropriate [31]. All statistical analysis was performed using SPSS (IBM SPSS Statistics 25). Statistically significant effects were assumed for *p* < 0.05.

## 3. Results

### 3.1. Animal Data

After coupling and confirmed pregnancy, the females were exposed to MP and BP as described in the Materials and Methods section. No remarkable changes in food and water consumption and no mortality were observed in any of the groups during gestation, nor were there any deaths in the descending population (F1 generation). The weight gain between the different groups up to the date of sacrifice (eight weeks after birth) was not significantly different (data not shown).

Regarding the relative mass of the organs, there was no change in the heart, liver, or kidneys (Table 1). Concerning the testes, there was a decrease in the relative mass for all groups compared to the CTRL group, with the difference being statistically significant for both BP concentrations and the higher MP concentration (Table 1). Seminal vesicles in all groups showed decreased mass, such as that observed in the testes; only the MP-100 group was not statistically different from the CTRL group (Table 1).

### 3.2. Respiration and Oxidative Phosphorylation of Isolated Mitochondria

**Figure 2:**
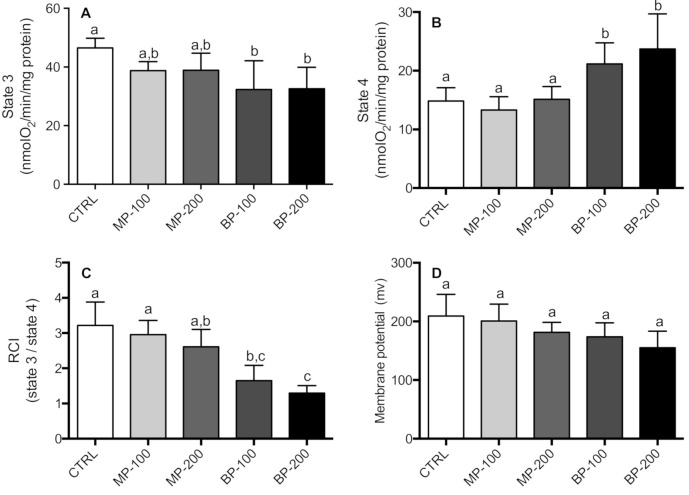
Oxygen consumption rates determined with mitochondria isolated from testicles of F1 generation rats. The parameters presented are respiratory state 3 (**A**), state 4 (**B**), respiratory control index (RCI) (**C**), and transmembrane potential (**D**). Mitochondria (0.8 mg/mL) were incubated at 30 °C in 1 mL of normal respiratory medium supplemented with rotenone (1.5 μg/mL). State 3 was obtained by the addition of 200 nmol of ADP in mitochondria to oxidize succinate (10 mM). State 4 was reached after total phosphorylation of ADP. The RCI was calculated as the ratio between the oxygen consumption rate in state 3 and state 4. Succinate-energized mitochondria ΔΨ was measured by means of a tetraphenylphosphonium-selective electrode. Freshly isolated mitochondria (1 mg) were incubated in 1 mL of the medium supplemented with 2 µM rotenone and 3 µM TPP^+^ and energized with 5 mM succinate. Results are expressed as nmol of oxygen consumed/min/mg of mitochondrial protein and are M ± SD of 7–10 independent experiments performed in duplicate. The values marked with the same letter are not statistically significant as determined by the Tukey’s post hoc test (*p* < 0.05).

The effect of exposure to MP and BP during gestation on mitochondrial respiration was also investigated. State 3 respiration in the presence of succinate (10 mM) and ADP (100 nmol) showed a decrease in all groups. However, only the groups exposed to BP were statistically different from the CTRL group (Figure 2A). State 4 showed an increase in the respiration rate for both BP groups. This difference was statistically significant compared to the MP and CTRL groups (Figure 2B). RCI showed a decrease in the MP and BP groups and was found to be concentration-dependent. However, only the BP groups were statistically different (Figure 2C). Regarding mitochondrial transmembrane potential, paraben treatment did not show any statistical difference from the CTRL group (Figure 2D).

**Figure 3:**
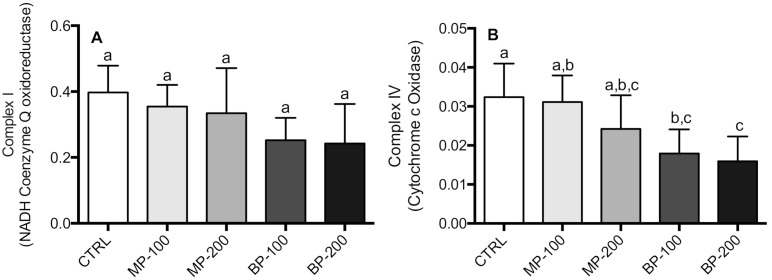
Activities of complexes I and IV of the mitochondrial electron transport chain of testes of F1 generation. The enzymatic activities are expressed as nmol oxidized NADH (**A**) and nmol cytochrome c oxidized (**B**). The results are M ± SD of 7–10 independent experiments performed in duplicate. The values marked with the same letter are not statistically significant as determined by the Tukey’s post hoc test (*p* < 0.05).

Mitochondrial electron transport chain complexes I and IV activities were also evaluated (Figure 3A,B). However, in complex IV, the differences were observed only in groups exposed to butylparaben showing a concentration-dependent decrease.

**Figure 4:**
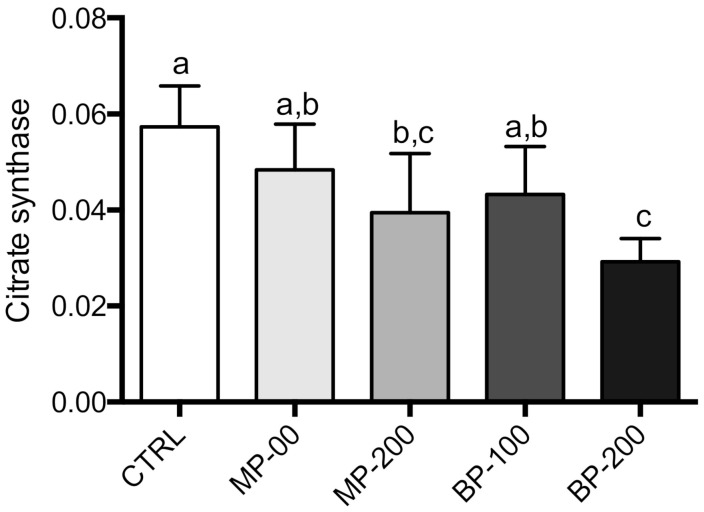
Citrate synthase activities in mitochondria isolated from testes of F1 generation. The results are M ± SD of 7–10 independent experiments performed in duplicate and expressed as nmol 2-nitro-5-thiobenzoic acid/min/mg protein. The values marked with the same letter are not statistically significant between groups as determined by the Tukey’s post hoc test (*p* < 0.05).

In the case of citrate synthase, we observed a statistically significant decrease for the highest paraben concentrations, with a decrease of about 50% in the BP-200 group compared to the CTRL group (Figure 4). This is a worrying result as it may indicate less mitochondrial content in this tissue.

**Figure 5:**
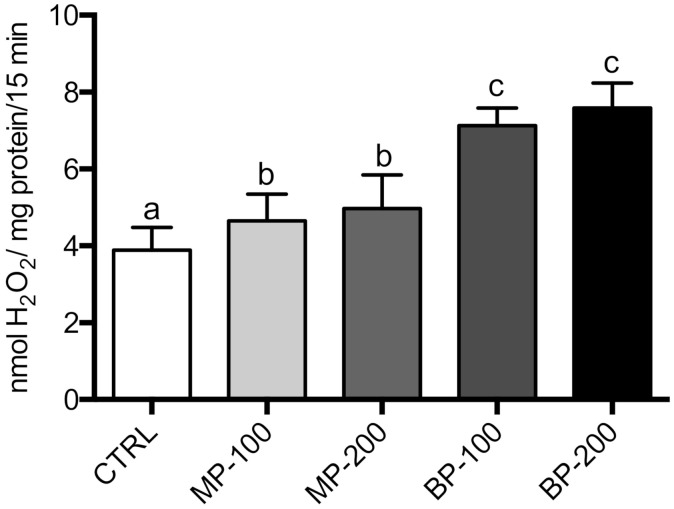
Production of hydrogen peroxide by functional mitochondria isolated from testes of F1 generation. The results are expressed as nmol H_2_O_2_/mg protein/15 min and are represented as M ± SD of 7–10 independent experiments performed with different mitochondrial fractions performed in duplicate. The values marked with the same letter are not statistically significant between groups as determined by the Tukey’s post hoc test (*p* < 0.05).

We also evaluated the production of mitochondrial ROS in the different groups through the formation of a homovanilic acid dimer whose conjugation was catalyzed by peroxidase in the presence of H_2_O_2_, as described in the Materials and Methods section. The production of H_2_O_2_ by mitochondria gives us an idea of the greater or lesser propensity for the occurrence of oxidative stress promoted by mitochondria. The results obtained (Figure 5) showed that the mitochondria isolated from the testes of the rats exposed to parabens during the gestation period presented a higher production of H_2_O_2_ than the rats of the CTRL group. In the BP group, this difference was highly significant and different from the group exposed to MP.

### 3.3. Oxidative Stress

**Figure 6:**
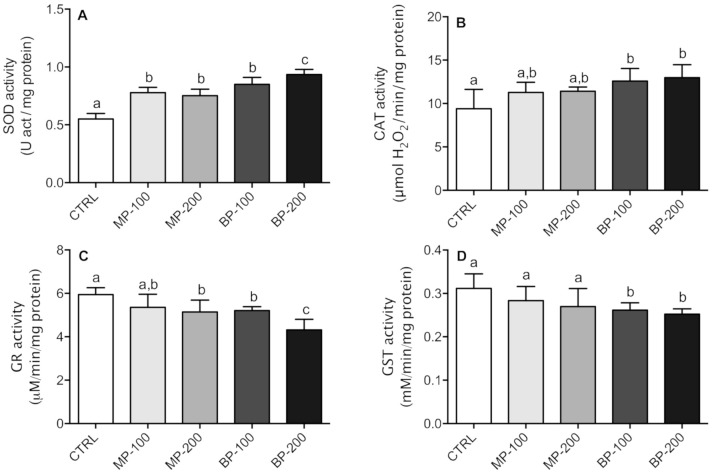
Activity of oxidative stress enzymes in testes of F1 generation rats: (**A**) superoxide dismutase (SOD), (**B**) catalase (CAT), (**C**) glutathione reductase (GR), and (**D**) glutathione *S*-transferase (GST). Results are M ± SD of 7–10 independent experiments. The values marked with the same letter are not statistically significant between groups as determined by the Tukey’s post hoc test (*p* < 0.05).

Paraben administration in pregnant animals during the entire gestation period induced significant changes in some antioxidant enzymes in the testes of the F1 generation, namely SOD, CAT, GR, and GST. All groups exposed to parabens showed an increase in SOD activity, which appeared to be concentration-dependent in the case of butylparaben (Figure 6A). Regarding CAT, only the groups exposed to butylparaben showed statistically significant differences compared to the CTRL group (Figure 6A).

In the case of GR, the groups exposed to the highest concentration of methylparaben showed a significant decrease, as did all the groups exposed to butylparaben. The BP-200 group presented differences relative to all the groups analyzed (Figure 6C). Regarding GST activity, only the groups exposed to butylparaben showed a statistically significant decrease, both with the CTRL group and the groups exposed to methylparaben (Figure 6D). These results clearly show that exposure to butylparaben interferes more significantly with the antioxidant system than methylparaben (Figure 6A–D).

**Figure 7:**
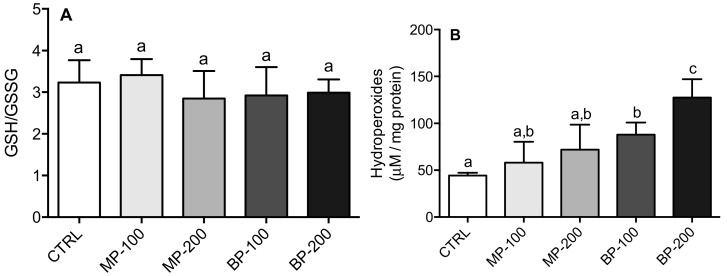
The glutathione (GSH)/glutathione disulfide (GSSG) ratio (**A**) and lipid hydroperoxide concentration (**B**) in the testes of the F1 generation rats. Results are M ± SD of 7–10 independent experiments. The values marked with the same letter are not statistically significant between groups as determined by the Tukey’s post hoc test (*p* < 0.05).

Despite the decrease in GR activity in the MP-200 group and both BP groups, there was no change in the GSH/GSSG ratio (Figure 7A). In the case of hydroperoxide formation (Figure 7B), both parabens had a concentration-dependent effect. However, only the BP groups had a statistically significant difference from the CTRL group. It was even statistically different from all the other groups at the highest concentration (200 mg/kg).

The SOD, CAT, GR, and GST activities were also determined in other organs, namely liver, kidney, heart, and seminal vesicles.

A one-way MANOVA was conducted to compare the effect of SOD, CAT, GR, and GST activities on other organs, namely liver, kidney, heart, and seminal vesicles. A significant multivariate effect was found (*p* < 0.001). Follow-up univariate analyses indicated no significant differences in SOD activity in the liver (*p* = 0.302) and heart (*p* = 0.128), whereas the differences were statistically significant in kidney and seminal vesicles (*p* < 0.05). Tukey’s post hoc test indicated significant differences for kidney between the MP-100 group and the CTRL group as well as MP-100 and BP-200 group, whereas there were significant differences between the BP-200 group and all other groups (MP-100 and CTRL as well as MP-100 and BP-100) for seminal vesicles (Tables S1–S4).

Follow-up univariate analyses indicated significant differences for all tested organs (*p* < 0.05) for CAT and GST activity. In CAT activity, Tukey’s post hoc test indicated significant liver differences between the 200 mg/kg groups (MP and BP) and the three other groups (CTRL, MP-100, and BP-100). Regarding the heart, all groups were different from the CTRL group, while in seminal vesicles, the BP groups were different from all the others. For liver GST activity, only the BP-200 group showed a difference compared to the MP-200 and BP-100 groups. In the kidney, both the MP-200 and BP-200 groups showed significant differences to the CTRL group. In the heart and seminal vesicles, only the BP-100 and BP-200 groups showed differences to the CTRL group (Tables S1–S4).

Regarding GR activity, follow-up univariate analyses indicated significant differences in the kidney, heart, and seminal vesicles (*p* < 0.05). In the kidney, Tukey’s post hoc test indicated that only the BP-100 group did not show significant differences to the CTRL group. In the heart, all groups were different from the CTRL group. The BP-100 and BP-200 groups were also different from the other groups and each other. In seminal vesicles, the BP-100 and BP-200 groups were both different from the CTRL group (Tables S1–S4).

Univariate analyses indicated significant differences in GSH/GSSG ratio in the liver (*p* < 0.05). Tukey’s post hoc test indicated that only the MP-100 group had no significant differences from the CRTL group as well as the MP-100 and BP-100 groups (Tables S1–S4).

The formation of hydroperoxides was not evaluated in the seminal vesicles due to a lack of materials. The results obtained for the heart, liver, and kidneys showed statistically significant differences in all organs (*p* < 0.05). Tukey’s post hoc test indicated significant differences in the liver between the BP-200 group and all other groups (CTRL, MP-100, and BP-100). In the kidney, only the BP-100 group was not different from the CTRL group, whereas for the heart, there were significant differences between all groups and the CTRL and between treatments (100 and 200 mg/kg) with MP and BP. In the heart, the BP-200 group showed differences compared to all other groups (Tables S1–S4).

## 4. Discussion

Parabens are a group of alkyl esters of *p*-hydroxybenzoic acid (PABA), which are widely used as preservatives in cosmetics, toiletries, food, and pharmaceuticals [17]. Thus, human beings are frequently exposed to these molecules, which may raise some concerns about health problems. Health concerns also result from the possibility of simultaneous consumption of synthetic molecules from other chemical families, which can potentiate adverse effects. Pollock et al. (2017) showed that, in vivo, BP and PP can interfere with the pharmacokinetics of bisphenol A and that BP can also modulate 17β-estradiol concentrations [32]. Therefore, it is important to consider simultaneous exposure to various chemicals when determining regulatory exposure limits. In this sense, numerous studies have been carried out in vitro and in vivo to evaluate the possible toxicity of parabens [10,13,33,34,35,36].

An in vivo study with pregnant rats exposed to BP by subcutaneous injection (100 or 200 mg/kg/day) did not affect the number of offspring or sex ratio. Nevertheless, the number of live births was significantly decreased with both BP concentrations [8]. We did not observe any difference regarding the number of offspring or effects on sex with the two concentrations of MP or BP used (100 or 200 mg/kg/day). We also did not observe any change in the average number of litters regarding the number of live births. The weight gain in males between the different groups up to the date of sacrifice was not different from the CTRL group, which is consistent with a study published by Leppert et al. (2020). However, this study showed that there was significant weight gain with BP exposure in females. This weight gain after birth was linked to higher fat levels due to the increased adipocyte size and lower lean mass, which could have resulted from an increase in food intake compared to the control mice [37]. In humans, paraben exposure was positively correlated with trimester gestational weight gain rate during pregnancy, especially during the first trimester [5].

The kidneys, heart, and liver weight did not present substantial alterations regarding the body mass. As for the reproductive organs, there was a decrease in the weight of testes and seminal vesicles, which confirms the results obtained by other authors [19]. A decrease in human sperm count and various sperm motility parameters have already been positively associated with the use of MP, EP, and BP, especially with hydroxylated metabolites [38].

Parabens have been identified in human placentas [39]. Fetus exposure is an aspect that must be analyzed as it is particularly susceptible to endocrine-disrupting agents [40]. After 6 h of exposure to BP, it can promote a significant increase in apoptosis in spermatogenic cells [41]. In vivo and in vitro studies have also demonstrated that treatment with BP induced rupture of vimentin filaments in Sertoli cells [33]. In isolated spermatogenic cells, the inducing effect of apoptosis via mitochondrial cytochrome c release has been reported [42]. These studies clearly showed that BP disturbingly affected the spermatogenesis process. Another worrying result was observed in pregnant women exposed to parabens, where changes in the expression of some cytokines were registered. Previous results suggest that exposure to various compounds, including butylparaben, during pregnancy may be related to oxidative stress and inflammation, which may influence birth outcomes and cause adverse health effects. However, further studies are needed to confirm these associations and the relationships between these compounds and adverse reproductive and birth outcomes [43]. Oral and subcutaneous exposure to rats with butylparaben resulted in offspring with raised concentrations of IL-1β, IL-6, and TNF-α in the brain tissues, suggesting that exposure to parabens alters cytokine expression on peripheral tissues. An adequate expression of cytokines is necessary for correct neurodevelopment and behavior, and any disturbances in the cytokine network can affect it. Thus, butylparaben causing an increase in TNF-a, IL-1b, and IL-6 in the brain tissues of puppies represents the first component of a disrupted neuroimmune network [44]. It has also been shown that exposure to hays and parabens can interfere with the pro- and anti-inflammatory system. Authors have also drawn attention to the need for future studies to better understand the complex mechanisms that link exposure to phenol and parabens to immunological disorders during pregnancy [45].

The mitochondria used in the present study were isolated from a mixture of somatic and germ cells. Given the active spermatogenesis in progress, the percentage of mitochondria will be predominantly obtained from germ cells, especially from spermatocytes and spermatids. In this study, mitochondrial respiration in state 3 and state 4 of the MP groups did not present significant alterations compared to the CTRL group. However, the BP groups showed statistically significant changes in both respiratory states, and in state 4, it was even different from the MP group at both the concentrations. As a result of this, the RCI significantly decreased in the groups exposed to BP, indicating a decrease in mitochondrial efficiency. Moreover, the increase in respiration of state 4 and a decrease in mitochondrial membrane potential (ΔΨ) are clear indicators of increased proton leakage [46], thus indicating less mitochondrial coupling.

The activities of complexes I–III did not show any differences (data not shown). Complex IV was significantly inhibited in the groups exposed to butylparaben (Figure 3), corroborating that the mitochondrial function was affected in the F1 generation.

Citrate synthase activity also showed a decrease, which is a function of chain length and paraben concentration, but the difference was significant only for the BP-200 group. This result is extremely relevant if we consider that this activity can be used as an indicator of mitochondrial content in tissues. We are not aware of any study that has shown a relationship between parabens and mitochondrial biogenesis. This result may indicate a decrease in mitogenesis in these cells [47], which will have significant implications in the reproductive capacity of these animals or decrease the energy available for the cell as it means inhibition of the Krebs cycle. Any decrease in mitochondrial biogenesis should be further confirmed by electron microscopy analysis to account for both the volume and the mitochondrial morphology itself.

In addition to this relevant aspect, we also observed that mitochondria isolated from the testes of animals exposed to parabens produced higher amounts of ROS. This ROS increase is a clear indicator of mitochondrial dysfunction due to exposure to parabens during the gestation period. It should be noted that mitochondrial ROS production was observed in mitochondria oxidizing succinate in the presence of rotenone. Therefore, the value may be underestimated as complex I and complex III are considered the main sites of ROS production in mitochondria. Samarasinghe et al. (2018) observed that human spermatozoa exposed to paraben increased ROS production [15]. In another study, the changes induced by parabens in the reproductive capacity of *Caenorhabditis elegans* were attributed to the induction of oxidative stress [48].

This study corroborates our findings related to oxidative stress because, except for the seminal vesicles where the quantification of hydroperoxides was not performed, all other organs presented an increase in hydroperoxide content, mainly in the BP-200 group, which is in agreement with the results obtained by Shah and Verma (2011) regarding the hepatotoxicity resulting from oral exposure to BP [10].

The real impact of parabens on reproduction and pregnancy in humans needs to be elucidated. Therefore, it is urgent to carry out studies to clarify the exact role of parabens in human male reproductive capacity. In several studies, urinary paraben concentration has been linked to sperm DNA damage and oxidative stress in pregnant women [15]. However, we are not aware of any study evaluating the implications of paraben use during pregnancy in different organs of the F1 generation apart from the male and female reproductive organs.

Our results showed that all organs suffered some modulation in the antioxidant enzyme systems resulting from MP and BP exposure during pregnancy. However, among the studied organs (liver, kidney, heart, and seminal vesicles presented in Supplementary Materials), the testicles and seminal vesicles appeared to be the most affected. Therefore, this seems to indicate that the reproductive organs are the most susceptible to paraben exposure during intrauterine development, which may decrease sperm motility and quality [38], thereby reducing fertility.

Our results showed that despite the cellular changes observed in some enzyme activity in the antioxidant system, this response would not be sufficient to prevent oxidative damage at the membrane level, as confirmed by the increase in hydroperoxide content. This result could be explained by the membrane accumulation of parabens, especially those of the long chain [48]. Based on current knowledge about the effect of parabens, it seems plausible that parabens interfere with multiple pathways and therefore have an in vivo multifactorial role. We may further claim that if paraben exposure, even in lower concentrations, continues during lactation, it could exacerbate the observed damages. Moreover, it is expected that these changes observed in the bioenergetics and antioxidant system in the F1 offspring may be more intense in older animals.

Despite some controversy regarding paraben toxicity, our study indicates that paraben exposure during the gestation period will promote changes in mitochondrial bioenergetics and the antioxidant capacity of young F1 generation males. However, it is essential to perform more studies to clarify these effects, and it will be interesting to conduct similar studies in older F1 generation.

## 5. Conclusions

We found evidence that paraben use during the gestation period can negatively affect mitochondrial function in testicles of the F1 generation, leading to increased ROS production and modulating the antioxidant system in different organs. This research demonstrates the transgenerational effects of parabens on testicular bioenergetic and associated oxidative stress, contributing to the understanding of male infertility in future generations.

## Figures and Tables

**Table 1:** Relative mass (g) of different organs of animals (mean (M) ± standard deviation (SD)) of different groups. The relative organ weights (organ weight/animal weight) in percentage. The results with different letters mean they are statistically different from each other (*p* < 0.05).

Groups	Heart	Liver	Right Kidney	Left Kidney	Right Testicle	Left Testicle	Seminal Vesicles
Control	0.34 ± 0.04 ^a^	3.56 ± 0.33 ^a^	0.34 ± 0.05 ^a^	0.36 ± 0.09 ^a^	0.74 ± 0.01 ^a^	0.75 ± 0.09 ^a^	0.33 ± 0.11 ^a^
Methyl 100	0.40 ± 0.08 ^a^	3.79 ± 0.49 ^a^	0.44 ± 0.12 ^a^	0.42 ± 0.06 ^a^	0.63 ± 0.06 ^a.b^	0.67 ± 0.08 ^a.b^	0.29 ± 0.10 ^a^
Methyl 200	0.43 ± 0.11 ^a^	3.89 ± 0.34 ^a^	0.40 ± 0.06 ^a^	0.41 ± 0.09 ^a^	0.60 ±0.01 ^b^	0.59 ± 0.14 ^b.c^	0.22 ± 0.06 ^b^
Butyl 100	0.43 ± 0.11 ^a^	3.69 ± 0.33 ^a^	0.43 ± 0.07 ^a^	0.42 ± 0.06 ^a^	0.58 ± 0.15 ^b^	0.56 ± 0.1 ^b.c^	0.20 ± 0.07 ^b^
Butyl 200	0.39 ± 0.07 ^a^	3.65 ± 0.46 ^a^	0.39 ± 0.05 ^a^	0.38 ± 0.05 ^a^	0.52 ± 0.12 ^b^	0.54 ± 0.09 ^c^	0.20 ± 0.06 ^b^

## Supplementary Materials

The following are available online at https://www.mdpi.com/2076-3921/9/12/1302/s1, Table S1: M, SD, and univariate effects of different antioxidant enzymes and GSH/GSSG ratio by concentration in the liver. The results with different letters mean they are statistically different from each other (*p* < 0.05). Table S2: M, SD, and univariate effects of different antioxidant enzymes and GSH/GSSG ratio by concentration in the kidney. The results with different letters mean they are statistically different from each other (*p* < 0.05). Table S3: M, SD, and univariate effects of different antioxidant enzymes and GSH/GSSG ratio by concentration in the heart. The results with different letters mean they are statistically different from each other (*p* < 0.05). Table S4: M, SD, and univariate effects of different antioxidant enzymes and GSH/GSSG ratio by concentration in the seminal vesicles. The results with different letters mean they are statistically different from each other (*p* < 0.05).
