# Supplementary material for: Use of Parabens (Methyl and Butyl) during the Gestation Period: Mitochondrial Bioenergetics of the Testes and Antioxidant Capacity Alterations in Testes and Other Vital Organs of the F1 Generation
Source: Antioxidants (Basel). 2020 Dec 18;9(12):1302. doi: 10.3390/antiox9121302 (PMC7766258; doi:10.3390/antiox9121302)
Supplement: Supplementary file 1 [file antioxidants-09-01302-s001.pdf]

antioxidants

Table S1. Means (M), standard deviations (SD) and univariate effects of the different antioxidant enzymes and GSH/GSSG ratio by concentration in the liver. The results with different letters mean that they are statistically different from each other ($p < 0.05$).

Liver							
	**CTRL**	**MP-100**	**MP-200**	**BP-100**	**BP-200**	**F**	***p***
**ENZYMES**	M ± SD	M ± SD	M ± SD	M ± SD	M ± SD		
**SOD**	1.51 ± 0.25 [a]	1.75 ± 0.23 [a]	1.64 ± 0.28 [a]	1.74 ± 0.16 [a]	1.64 ± 0.29 [a]	1.267	0.302
**CAT**	939.43 ± 157.90 [a]	829.75 ± 115.53 [a]	669.21 ± 154.64 [b]	826.82 ± 152.03 [a]	714.80 ± 72.99 [b]	5.599	0.002
**GR**	1.55 ± 0.30 [a]	1.48 ± 0.28 [a]	1.20 ± 0.36 [a]	1.28 ± 0.25 [a]	1.21 ± 0.23 [a]	2.493	0.061
**GST**	0.25 ± 0.05 [a,b]	0.22 ± 0.03 [a,b]	0.21 ± 0.04[a]	0.21 ± 0.01 [a]	0.26 ± 0.02[b]	3.842	0.011
**GSH/GSSG**	6.21 ± 1.05 [a]	5.79 ± 0.53 [a,b]	4.54 ± 0.62 [c]	5.08 ± 0.65 [b,c]	4.51 ± 0.46 [c]	9.485	<0.001
**Hydroperoxides**	65.49 ± 6.09 [a]	72.25 ± 7.21 [a]	77.10 ± 6.45 [a]	74.47 ± 10.34 [a]	94.55 ± 9.72 [b]	8.831	<0.001

Table S2. Means (M), standard deviations (SD) and univariate effects of the different antioxidant enzymes and GSH/GSSG ratio by concentration in the kidney. The results with different letters mean that they are statistically different from each other ($p < 0.05$).

Kidney							
	**CTRL**	**MP-100**	**MP-200**	**BP-100**	**BP-200**	**F**	***p***
**ENZYMES**	M ± SD	M ± SD	M ± SD	M ± SD	M ± SD		
**SOD**	1.84 ± 0.18 [a]	1.54 ± 0.16 [b]	1.65 ± 0.14 [a,b]	1.77 ± 0.19 [a,b]	1.84 ± 0.12 [a]	4.692	0.005
**CAT**	6499.8 ± 932.9 [a,b]	6758.0 ± 1716.5 [b]	6275.0 ± 1126.4 [a,b]	4351.0 ± 1279.6 [a]	4514.7 ± 1442.1 [a,b]	4.526	0.007
**GR**	2.68 ± 0.54 [a]	3.64 ± 0.52 [b]	4.85 ± 0.82 [c]	3.31 ± 0.53 [a,b]	3.75 ± 0.94 [b]	13.055	<0.001
**GST**	0.58 ± 0.15 [a]	0.55 ± 0.09 [a]	0.77 ± 0.07 [b]	0.66 ± 0.09 [a,b]	0.83 ± 0.11 [b]	8.005	<0.011
**GSH/GSSG**	3.96 ± 0.61 [a]	3.93 ± 0.39 [a]	3.83 ± 0.37 [a]	4.14 ± 0.56 [a]	4.09 ± 0.39 [a]	0.422	0.791
**Hydroperoxides**	81.1 ± 14.6 [a]	107.9 ± 9.4 [b,c]	117.6 ± 8.2 [b]	98.8 ± 12.8 [a,c]	101.3 ± 6.8 [b,c]	7.803	0.001

Table S3. Means (M), standard deviations (SD) and univariate effects of the different antioxidant enzymes and GSH/GSSG ratio by concentration in the heart. The results with different letters mean that they are statistically different from each other ($p < 0.05$).

**Heart**							
	**CTRL**	**MP-100**	**MP-200**	**BP-100**	**BP-200**	**F**	***p***
**ENZYMES**	M ± SD	M ± SD	M ± SD	M ± SD	M ± SD		
**SOD**	1.6 ± 0.3 [a]	1.5 ± 0.2 [a]	1.3 ± 0.3 [a]	1.6 ± 0.3 [a]	1.3 ± 0.3 [a]	1.952	0.128
**CAT**	6.40 ± 0.90 [a]	9.29 ± 2.48 [b]	10.85 ± 1.07 [b,c]	11.89 ± 1.12 [c,d]	13.79 ± 1.28 [d]	25.041	<0.001
**GR**	0.0005 ± 0.0003 [a]	0.0014 ± 0.0002 [b,c]	0.0011 ± 0.0002 [b]	0.0016 ± 0.0004 [c]	0.0022 ± 0.0004 [d]	31.918	<0.001
**GST**	0.0086 ± 0.0012 [a]	0.0085 ± 0.0014 [a]	0.0077 ± 0.0015 [a,b]	0.0062 ± 0.0011 [b]	0.0060 ± 0.0012 [b]	7.822	<0.001
**GSH/GSSG**	5.34 ± 0.62 [a]	4.86 ± 0.81 [a]	5.27 ± 0.32 [a]	5.19 ± 0.66 [a]	5.29 ± 0.198 [a]	0.683	0.610
**Hydroperoxides**	80.76 ± 5.23 [a]	89.47 ± 1.36 [b]	99.01 ± 7.08 [c]	94.23 ± 4.72 [b,c]	123.99 ± 4.89 [d]	63.366	<0.001

Table S4. Means (M), standard deviations (SD) and univariate effects of the different antioxidant enzymes and GSH/GSSG ratio by concentration in the seminal vesicles. The results with different letters mean that they are statistically different from each other ($p < 0.05$).

**Seminal vesicles**							
	**CTRL**	**MP-100**	**MP-200**	**BP-100**	**BP-200**	**F**	***p***
**ENZYMES**	M ± SD	M ± SD	M ± SD	M ± SD	M ± SD		
**SOD**	1.14 ± 0.09 [a,c]	0.81 ± 0.18 [b]	1.029 ± 0.27 [a,b]	1.36 ± 0.14 [c]	1.75 ± 0.09 [d]	31.635	<0.001
**CAT**	1.22 ± 0.09 [a]	1.15 ± 0.11 [a]	1.12 ± 0.21 [a]	0.89 ± 0.15 [b]	0.74 ± 0.22 [b]	12.437	<0.001
**GR**	7.22 ± 1.89 [a]	7.50 ± 2.17 [a]	5.46 ± 0.69 [a,b]	4.09 ± 0.98 [b]	3.99 ± 1.07 [b]	10.232	<0.001
**GST**	0.012 ± 0.0011 [a]	0.011 ± 0.0023 [a]	0.012 ± 0.0022 [a,b]	0.009 ± 0.0024 [b,c]	0.0078 ± 0.0025 [c]	6.600	<0.001
**GSH/GSSG**	5.29 ± 1.69 [a]	4.99 ± 0.96 [a]	5.07 ± 0.99 [a]	5.19 ± 1.11 [a]	4.61 ± 1.28 [a]	0.452	0.771
